# Psychological violence against general practitioners and nurses in Chinese township hospitals: incidence and implications

**DOI:** 10.1186/s12955-018-0940-9

**Published:** 2018-06-05

**Authors:** Peng Li, Kai Xing, Hong Qiao, Huiying Fang, Hongkun Ma, Mingli Jiao, Yanhua Hao, Ye Li, Libo Liang, Lijun Gao, Zheng Kang, Yu Cui, Hong Sun, Qunhong Wu, Ming Liu

**Affiliations:** 1 Department of Health Policy and Hospital Management, School of Public Health, Harbin Medical University, Harbin, 150081 China; 2 Endocrine and Metabolic Diseases, the 2nd Affiliated Hospital of Harbin Medical University, Harbin, 150081 China; 3 Department of Health Management, Harbin Medical University, Harbin, 150081 China; 4 Institute of Quantitative and Technical Economics, Chinese Academy of Social Science, Beijing, 100000 China; 5 Department of Social Medicine, School of Public Health, Harbin Medical University, Harbin, 150081 China; 6 Department of Medical Demography, School of Public Health, Harbin Medical University, Harbin, 150081 China; 7 Otorhinolaryngology, the 2nd Affiliated Hospital of Harbin Medical University, Harbin, 150081 China

**Keywords:** Psychological violence, Risk factors, Township hospitals, Workplace violence

## Abstract

**Background:**

International reports indicating that around 10–50% of health care staff are exposed to violence every year; in certain settings, this rate might reach over 85%. Evidence has shown that people who experience psychological violence are seven times as likely to be victims of physical violence. Although there have been numerous studies on WPV in general hospitals, there is no consensus regarding the current status of psychological violence directed at health care workers in township hospitals in China. The purpose of this study was to estimate the prevalence and the risk factors of psychological violence in Chinese township hospitals.

**Methods:**

A retrospective cross-sectional survey of township hospitals general practitioners and general nurses was conducted in Heilongjiang Province, China.Descriptive analyses and binary logistic regression analysis were used to estimated the prevalence and the risk factors of psychological violence.

**Results:**

Regardless of whether the assessment period was the past 12 months, past 36 months, or during their entire career,GPs and nurses reported that verbal abuse was the most common type of psychological violence (28.05, 30.28, 38.69 and 40.45%, 43.86, 54.02%).The main perpetrator was patients’ relatives. Most participants responded to violence with “pretend nothing happened”, 55.63% of GPs and 62.64% of nurses reported that the perpetrator received no punishment. Around 47.62% of respondents reported that their workplace had no procedures for reporting violence. When workplaces did have a reporting system, 57.73% knew how to use them. Only 36.98% had training in managing aggression and violence. General nurses, individuals 35 years or younger, those with higher professional titles and who work in shifts are at greater risk of psychological violence.

**Conclusions:**

Our results indicate a high prevalence of psychological violence in Chinese township hospitals, which can no longer be ignored. Effective measures should be taken to prevent and respond to workplace violence(WPV), especially psychological violence.

**Trial registration:**

(Project Identification Code: HMUIRB20160014), Registered May 10, 2016.

## Background

Workplace violence (WPV) is defined as “incidents where staff are abused, threatened or assaulted in circumstances related to their work, including commuting to and from work, involving an explicit or implicit challenge to their safety, well-being or health” [1]. Health care workers are 16 times as likely to experience WPV as are other workers, while nurses in particular are three times as likely [2]. WPV against health care workers is common across different countries [3–5], with international reports indicating that around 10–50% of health care staff are exposed to violence every year; in certain settings, this rate might reach over 85% [6].

According to the World Health Organization (WHO), WPV can be categorized as physical, psychological (emotional), sexual, and racial [7]. Physical and psychological violence are both common, but psychological violence appears to be more so [8]. Psychological violence can be defined as the intentional act against a person or collective force that results in physical, mental, spiritual, moral, and social harm, including insults, threats, attacks, verbal abuse, and harassment [1]. This definition of psychological violence, created by the WHO, is what we employ in the present study.

More specifically, we operationalize psychological violence as verbal abuse, Yi Nao, threats, and sexual harassment. Health care workplaces in China are unique given the involvement of Yi Nao, which is literally defined as “health care disturbance.” Hesketh and Wu described Yi Nao as gangs consisting “largely of unemployed people with a designated leader. They threaten and assault hospital personnel, damage facilities and equipment, and prevent the normal activities of the hospital.” More broadly, Yi Nao can mean any medical or hospital disturbance created by a group of people—such as patients, patients’ families, relatives, or Yi Nao gang members hired by patients or their families—who gather at hospitals involved in disputes with patients for actual or perceived medical malpractice. A 2006 survey of 270 tertiary hospitals reported that over 73% of the participating hospitals had experienced Yi Nao [9]. The aim of Yi Nao is typically to force the hospital to reduce costs or obtain compensation. When financial benefit is their main target, these gangs use extreme acts or criminal behaviors in a devious manner, often avoiding physical violence that would lead to formal punishment under the law; instead, they tend to threaten or abuse health care workers verbally to pressure hospitals into accepting their demands.

Psychological and physical violence among health care workers is associated with decreased job satisfaction, increased occupational strain, and poor patient care outcomes [10–12]. Additionally, WPV negatively influences health care workers’ organizational commitment [13]. Moreover, the consequences for the patients and entire facility are serious because the health care workers who perceive themselves at risk of violence are likely to offer poorer quality care and treatment, which in turn has adverse outcomes for patients [14]. Sometimes psychological or verbal abuse has more severe consequences than acts of physical violence.

Evidence has shown that people who experience psychological violence are seven times as likely to be victims of physical violence [15]. Studies set in America in 2004 and 2015 have shown that verbal abuse is the most frequent type of violence reported by physicians and nurses (39–99%), with physical violence being experienced by only 1–11% [16–18]. Furthermore, in a study in Pakistan, more than two-thirds of the respondents (*n* = 121/164, 73.8%) were victims of violence in the preceding 12 months, with verbal abuse (*n* = 104/121, 86%) being the main type of aggression [19]. In Jordan, the prevalence of verbal abuse by patients and visitors was 63.9%, while for physical abuse, 7.2% was committed by patients and 3.1% by visitors [20]. Approximately 30% of hospital staff in central Taiwan reported having experienced only verbal abuse [21]. In both the private and public sectors in Hong Kong, non-physical violence was found to occur more frequently than physical violence; furthermore, there is a reported lack of preparedness of many organizations in dealing with violence [22]. In Italy, around one-tenth of workers have reported some form of physical assault in the workplace, while as many as one-third have been exposed to non-physical violence in the previous year. Nurses and physicians were found to be the most vulnerable occupations [23].

In a past study on hospital violence in China, the incidence of violence in Chinese hospitals reached as high as 95%, indicating that the physical and verbal abuse of medical staff is common [24]. The frequency of psychological violence has also been shown to be higher than that of physical violence—indeed, although violence and aggression towards nurses are frequent, non-physical violence appears far more prevalent (71.9%) than physical violence (7.8%). Around 24% of respondents in a Chinese study reported suffering from non-physical violence in relation to Yi Nao [25]. Furthermore, in one survey of the general hospitals in Guangdong Province, the prevalence of psychological violence was 49.12%, while the prevalence of physical violence was 15.36%. Men were found to be more vulnerable to violence, whereas women were more vulnerable to non-physical violence [26].

In China, township hospitals are comprehensive health administration and medical institutions that provide basic rural health services for people living in these towns. They are regarded as the hubs of the rural tertiary health care system. China’s new round of healthcare reform and its 12th Five-Year Plan for the medical service system have focused on improving and strengthening township-level health facilities. The main goal is to reduce the number of common and frequently occurring diseases. General practitioners (GPs) are central to the health care teams of township hospitals in the future because of the particular status and working characteristics of township hospitals in China [27]. According to the China Health Statistics Yearbook 2013, a report by the Ministry of Health of China [28], China had 37,097 township hospitals, of which there were 996 in Heilongjiang province, and these had 2081 GPs and 3616 registered nurses.

Although there have been numerous studies on WPV in general hospitals, there is no consensus regarding the current status of psychological violence directed at health care workers in township hospitals in China.What is the level of psychological violence in China’s rural GPs and nurses? Does it happen to have the same risk factors as other countries? Can we directly cite the other findings to deal with the psychological violence in township hospitals in China? With these questions, we started our research. The specific purposes of this study are to identify the prevalence and severity of psychological violence against GPs and general nurses in township hospitals in Heilongjiang province, northeastern China, and to identify the risk factors contributing to psychological violence in these hospitals.

## Methods

A retrospective cross-sectional survey of general practitioners and general nurses was conducted in Heilongjiang Province, China. In 2012, Heilongjiang had a population of 38.1 million and 996 township hospitals. We randomly selected 90 township hospitals in Heilongjiang Province. Permission to administer the survey was obtained from all 90 township hospitals. The collected data were used to publish an article about physical violence in 2015 [29].

### Data collection

The survey was conducted from September to November 2014, and access was negotiated through the participants’ supervisors in every study hospital. An anonymous, self-administered paper questionnaire was distributed to each participant. The questionnaire also included a notification letter and return envelope; the study purpose and rights of the healthcare workers regarding participation were declared in the letter. The participants had 7 days to complete the questionnaire; once they had done so, they placed the completed questionnaire into the return envelope and placed the envelope into a box in the department manager’s office to ensure privacy and anonymity. The data collected were secured in a locked room that could only be accessed by research personnel. In this survey, all doctors and nurses (N = 990) of the selected hospitals were approached and a total of 990 questionnaires were distributed .

### Questionnaire

The questionnaire used was developed through a literature review and by modifying a questionnaire developed in 2003 by a joint program of the International Labour Office (ILO), International Council of Nurses, WHO, and Public Services International [30]. First, we formally obtained documented permission to use the questionnaire from the ILO and WHO. It was then translated into Mandarin Chinese and back-translated into English to verify the accuracy of the Mandarin version. Subsequently, the questionnaire was modified to fit our study objectives and the township hospital context in China. For example, Yi Nao was included as part of the items on psychological violence because it is unique to WPV in China. The content validity was determined by a panel of 18 healthcare-related experts throughout China, who were asked to assess the questionnaire in terms of its clarity, relevance, comprehensiveness, and sensitivity to Chinese culture. After revision by the expert committee, the questionnaire was administered to 30 participants as a pre-test. All of these individuals were subsequently excluded from the study. Further modifications were taken as per these individuals’ feedback. For all questions, the Cronbach’s alpha coefficient was 0.86. The questionnaire was then back translated to English to verify the accuracy of the Mandarin version.

The questionnaire was divided into four sections: (1) the demographic characteristics of the respondents and workplace data; (2) physical violence, including prevalence of physical violence, and the demographic characteristics of perpetrators, attack time, attack tools, and consequences; (3) psychological violence, including prevalence, response of healthcare workers, and workers’ methods of dealing with psychological violence; and (4) organizational measures, including incident reporting, supervisor support, and training programs. As this study focused on psychological violence, we used data only from sections “Background, Results, and Discussion”. Our questionnaire contains a total of 63 questions and the expected completion time is 10–15 min.

### Data analysis

The data were coded in EpiData, and analyzed using IBM SPSS Statistics 19.0 (IBM Corp., Armonk, NY). Descriptive analyses were used to address the study objectives. Binary logistic regression analysis was used to assess the potential associations between exposure to psychological violence in general (yes/no) and respondents’ characteristics, including age, gender, years of experience, educational level, occupation, professional title, and shift work status. Through variable selection (criteria: independent variables were entered and excluded from a binary regression model at *p* < 0.05), we entered the variables that meet the requirements into the binary logistic regression model. Odds ratios (ORs) and 95% confidence intervals (CIs) were calculated; *p* < 0.05 was considered statistically significant.

## Results

Of the 840 respondents(response rate = 84.8%), 442 were GPs and 398 were general nurses. Only valid responses and percentages were included. Both descriptive and binary logistic regression analyses are presented below.

### Demographic characteristics of respondents

A summary of these characteristics are shown in Table 1.

**Table 1:** Demographic characteristics of the respondents (N= 840)

Characteristics	Number	Percent
Gender		
Male	442	52.62
Female	398	47.38
Age		
< 35	140	16.67
35–45	426	50.71
> 45	274	32.62
Years of experiences		
*< 10*	116	13.81
*10–20*	394	46.90
*> 20*	330	39.29
Education		
Postgraduate	8	0.95
Undergraduate	380	45.24
College	348	41.43
Technical secondary school education and below	104	12.38
Professional Title		
Senior	170	20.24
Intermediate	410	48.81
Junior	192	22.86
No title	68	8.09
Occupation		
General practitioner	442	52.62
General nurse	398	47.38
Work in shifts		
Yes	385	45.83
No	455	54.17

### Prevalence of psychological violence

The type of violence suffered by those who suffer psychological violence is not exclusive.Due to the fact that some respondents worked less than 36 months in our survey, the number of respondents during the past 36 months was less than 840. Whether the assessment period was the past 12 months, past 36 months, or during their entire career, GPs and nurses reported that verbal abuse was the most common type of psychological violence (38.69, 54.02%; 30.28, 43.86 and 28.05%, 40.45%), followed by Yi Nao (23.08, 29.15%; 17.20, 20.10 and14.93%, 19.35%) and threats (20.36, 27.64%; 16.74, 22.98 and13.80%, 19.60%). These are shown in Table 2.

**Table 2:** Prevalence of psychological violence

Violence type	During career				During past 36 months				During past 12 months			
	GPs (442)		Nurses (398)		GPs (436)		Nurses (383)		GPs (442)		Nurses (398)	
	N	%	N	%	N	%	N	%	N	%	N	%
Verbal abuse	171	38.69	215	54.02	132	30.28	168	43.86	124	28.05	161	40.45
Yi Nao	102	23.08	116	29.15	75	17.20	77	20.10	66	14.93	77	19.35
Threat	90	20.36	110	27.64	73	16.74	88	22.98	61	13.80	78	19.60
Verbal sexual harassment	68	15.38	104	26.13	48	11.01	75	19.58	45	10.18	57	14.32
Sex harassment	38	8.60	62	15.58	32	7.34	44	11.49	27	6.11	37	9.30
Any type of violence	198	44.80	229	57.54	164	37.61	207	54.04	153	34.62	180	45.23

### Perpetrators of psychological violence and health care workers’ responses and methods of dealing with psychological violence

Of the 333 victims(during past 12 months), GPs and nurses reported that the main perpetrator were patients’ relatives (48.72, 52.54%), followed by patients (37.82, 32.20%). Additionally, few colleagues (0.64, 5.09%) and superiors (1.28, 4.52%) were reported as the perpetrator. As responses to psychological violence, 50.00% of the GPs and 37.30% of the nurses pretended nothing happened, 24.32% of the GPs and 28.11% of the nurses took no measures. About more than half of the victims considered these violent incidents as preventable. In the majority of incidents reported by respondents (GPs(57.05%), nurses(61.58%)), the perpetrator received no punishment. These are shown in Table 3.

**Table 3:** Perpetrators of psychological violence and health care workers’ response to and method of dealing with psychological violence (*N* = 333

	General practitioner		Nurse	
Perpetrator	N(156)	%	N(177)	%
Patient	59	37.82	57	32.20
Patient’s relative	76	48.72	93	52.54
Colleague	1	0.64	9	5.09
Supervisor	2	1.28	8	4.52
Others	18	11.54	10	5.65
Respond to the incident(Multiple choice)	N(410)	%	N(497)	%
No measures to take	36	24.32	52	28.11
Pretend nothing happened	74	50.00	69	37.30
Told the person to stop	45	30.41	52	27.66
Told friends/family	66	44.59	68	36.76
Sought counseling	18	12.16	19	10.11
Told a colleague	56	37.84	77	41.62
Transferred to another position	20	13.51	25	16.89
Reported it to a senior staff member	56	37.84	77	41.62
Completed incident/accident form	9	6.08	19	10.27
Pursued prosecution	15	10.14	16	8.64
Completed a compensation claim	7	4.73	9	4.86
Sought help from the union	8	5.41	14	7.57
The incident could have been prevented	N(156)	%	N(177)	%
Yes	88	56.41	105	59.32
No	68	43.59	72	40.68
Consequence for the perpetrator	N(156)	%	N(177)	%
None	89	57.05	109	61.58
Verbal warning issued by hospital managers	49	31.41	56	31.64
Stopping treatment	11	7.05	7	3.96
Reported to police	7	4.49	5	2.82

### Policy, procedures, and intervention strategies against workplace violence

Of the 840 respondents,around 47.62% of respondents (*n* = 400) reported that their workplace did not have procedures for reporting WPV. Where there was a reporting system, only 57.73% (*n* = 254) of respondents knew how to use it. Furthermore, 55.00% (*n* = 462) of respondents said that there was no incentive to report workplace violence. Only 39.17% reported having training in managing aggression and violence, and a total of 54.05% respondents (*n* = 454) reported that there were no specific measures for dealing with psychological violence in their workplace. All of the rates are presented in Table 4.

**Table 4:** Policy, procedures, and intervention strategies against workplace violence

	Number	Percent
Procedures for the reporting of violence in your workplace		
Reporting only if suffered physical injury	256	30.48
Reporting as long as suffered verbal threat	184	21.90
No	400	47.62
Know how to use them if have the procedures(*N* = 440)		
Yes	254	57.73
No	186	42.27
Encouragement to report workplace violence		
Yes	378	45.00
No	462	55.00
Train in the management of aggression and violence		
Yes	329	39.17
No	500	59.52
Data lost	11	1.31
Specific measures to deal with the psychological violence		
Yes	248	29.52
no	454	54.05
Do not know	138	16.43

### Binary logistic regression analysis

According to the logistic regression analyses, age, occupation, and professional title of respondents were found to have significant associations with exposure to psychological violence in general. More specifically, the odds of psychological violence were lower respondents who were 35 and 45 years (OR = 0.423, 95% CI = 0.280, 0.639), and 45 years and older (OR = 0.484, 95% CI = 0.313, 0.750) compared with those who were < 35 years old. Regarding occupation, compared to GPs, the odds of experiencing psychological violence were higher among general nurses (OR = 1.787, 95% CI = 1.330, 2.402). Respondents with lower professional titles had lower odds of being victims of psychological violence compared to respondents with higher professional titles (OR = 0.632, 95% CI = 0.541, 0.739). Finally, the odds of psychological violence were lower among those not working in shifts compared to those engaged in shift work (OR = 0.613, 95% CI = 0.455,0.826). All results are presented in Table 5.

**Table 5:** Risk factors associated with psychological violence among GPs and general nurses in township hospitals in Heilongjiang province (binary logistic regression model results)

Characteristics		Psychological violence (*n* = 333)											
		Univariate analysis						logistic regression(initial model)			logistic regression(final model)		
		*Yes*		*No*		*X* ^*2*^	*p*	*ORs*	*95% CIs*	*p*	*ORs*	*95% CIs*	*p*
		*n*	%	*n*	%								
Gender	Male	200	45.25	242	54.75	0.000	0.995	1.00	Reference				
	Female	180	45.23	218	54.77			0.809	(0.586,1.116)	0.379			
Age	< 35	73	51.41	69	48.59	9.922	0.007	1.00	Reference		1.00	Reference	
	35–45	156	36.97	266	63.03			0.423	(0.280, 0.639)	< 0.001	0.473	(0.283, 0.832)	0.006
	> 45	104	37.68	172	62.32			0.484	(0.313, 0.750)	0.001	0.544	(0.333, 0.956)	0.009
Years of experiences	< 10	49	41.53	69	58.47	1.160	0.560	1.00	Reference				
	10–20	147	37.69	243	62.31			0.640	(0.380,1.079)	0.094			
	> 20	137	41.27	195	58.73			0.445	(0.024–8.187)	0.498			
Education	Postgraduate	4	50.00	4	50.00	14.057	0.003	1.00	Reference				
	Undergraduate	135	35.71	243	64.29			0.645	(0.146,2.848)	0.562			
	College	158	45.14	192	54.86			1.136	(0.260,4.969)	0.866			
	Technical secondary school education and below	28	26.92	76	73.08			0.708	(0.156,3.215)	0.655			
Occupation	General Practitioner	156	35.29	286	64.71	7.373	0.007	1.00	Reference		1.00	Reference	
	General nurse	177	44.47	221	55.53			1.787	(1.330, 2.402)	0.001	1.832	(1.520, 2.603)	0.001
Professional title	Senior	102	58.62	72	41.38	33.033	< 0.001	1.00	Reference		1.00	Reference	
	Intermediate/Junior/No title	231	34.68	435	65.32			0.632	(0.541, 0.739)	< 0.001	0.680	(0.571, 0.810)	< 0.001
Work in shifts	Yes	169	43.90	216	56.10	5.374	0.020	1.00	Reference		1.00	Reference	
	No	164	36.04	291	63.96			0.613	(0.455,0.826)	0.001	0.675	(0.506,0.901)	0.008

## Discussion

In terms of the prevalence of psychological violence, our findings are similar to those reported from other countries, which have shown that verbal abuse is the most frequent type of violence [16–24]. Most pressingly, the results indicate that most health care workers suffer from verbal abuse, which suggests that there is an urgent need for policymakers or hospital managers to develop responses.

Arguably the most interesting finding in our study is that health care workers with higher level professional titles had higher odds of psychological violence, which has rarely been found in previous literature. Why is this the case? We suggest several reasons. First, health care workers with higher-level titles are often in contact, during their medical work, with patients or their families who are seriously ill. As such, when the effects of their treatment fall short of the patients’ and families’ expectations, these parties might blame the doctors, thus further triggering psychological violence. Second, those healthcare workers with higher titles might be involved in larger health care issues and medical disputes than those with lower titles in their daily work. Thus, they would have a greater likelihood of suffering from psychological violence.

General nurses were most exposed to psychological violence. First of all,nurses are more likely to encounter aggressive behavior because they tend to communicate and interact more with patients and their families than GPs. Second, according to the 2013 China Health Statistics Yearbook, the number of health workers in township hospitals in Heilongjiang Province in 2013 reached 2.26: 1 [28].This means that nurses in township hospitals have more work to do in their day-to-day work than doctors do. High workloads make them unable to fully meet the service needs of patients during the limited working hours, but also more prone to work under high pressure errors, resulting in patient dissatisfaction with the work of nurses. Furthermore, they frequently work at nights, have higher stress and workloads, and lack good management policies and support [31–34]. WPV has been found to be associated with stress and work strain; this connection is believed to be circular, as work strain and stress can be causes of WPV, which in turn leads to greater work strain and stress. Worse still, the increased negative stress leads to a greater likelihood of not only WPV, but also burnout, suicide, and even murder. Notably, the directional relationship of stress to violence is usually mediated by various factors, while the relationship of violence to stress is direct [35, 36]. Our study also found that health care workers who work in shifts show greater odds of psychological violence. We suspect that stress is implicated in this result as well: namely, those who often work in shifts might have higher stress levels and workloads, thus increasing the likelihood of WPV.

The logistic regression analysis also revealed that respondents of younger age had greater odds of experiencing psychological violence. Other studies have provided evidence that as health care workers’ age increases, the frequency of experiencing violence decreases [29, 37, 38].

In our study, we found some risk factors for psychosocial violence among healthcare workers in township hospitals in China.However, in the case of multiple risk factors clustering in one person (for example, a younger nurse with a shift experience a greater chance of psychological violence than a township health worker with only one risk factor experiences psychological violence.Interestingly, in practice, older doctors tend to have a higher professional title. In our study, doctors with high professional titles were found to have a higher risk of psychological violence. Therefore, in this case, the probability of their being subjected to psychological violence remains to be studied.

The present study has shown that patients’ families are the main source of psychological violence. Previous studies have similarly reported that 64.52 to 98.8% of aggressors are patients’ relatives [39–41]. This might be because, first, patients’ relatives tend to experience considerable stress due to economic, spiritual, and even social factors related to their family member’s illnesses. Furthermore, when they have high expectations for treatment and lack sufficient understanding of disease severity, they might feel increasingly helplessness and become discontented with staff, thus leading them to commit WPV [42]. Second, there might be miscommunication between patients’ families and health care staff, especially nurses, which suggests the necessity of improving the quantity and quality of nurses’ communication with patients and families.

In conclusion, through our research, we found some risk factors for health care workers who are more susceptible to psotection of these groups. For example, first of all, it is necessary to have sufficient financial support and safety facilities, in particular to strengthen the human resources provision of rural hospitals. Second, when it comes to helping township hospitals in tertiary hospitals in cities in China, the content of psychological violence prevention and treatment can be increased. In addition, in view of the patient’s sexual harassment of female health workers, we believe that early education and prevention are effective ways to solve such problems. For this reason, medical undergraduate and young doctors should train and inform about sexual harassment and how to deal with sexual harassment [43].

Notably, 55.63% of GPs and 62.64% of nurses referred that the perpetrators did not receive any kind of punishment. This should be a matter of concern, especially because evidence shows that WPV usually results in short- and long-term effects on victims’ physical and psychological state, and even their professional performance [44, 45]. Other studies [46, 47] have shown that individuals who experience psychological violence, and endure feelings/symptoms over time, might be at risk for adverse mental health outcomes such as acute stress disorder or post-traumatic stress disorder.

Why does most of the psychological violence of health care workers have a lower reporting rate? One of the reasons for underreporting is the assessment of the seriousness of health care workers about the violence they have suffered. If the victim considers that some issues of psychological violence are not particularly serious, they may not report. Ownship health workers may treat less-severe psychological violence (such as light verbal abuse) as part of the job and will not report such incidents [48]. If the victims thought that the problem was not serious, they might not report it. Second, this finding might be related to the fact that most perpetrators did not receive any kind of punishment. In other words, workers might have thought that responding to the incident would be of no use. Finally, more than half of the respondents said that their hospitals did not have specific measures for dealing with psychological violence and did not encourage the reporting of WPV in our study, which might have led victims to choose to remain silent. To solve this issue, a priority for hospital leaders would be paying greater attention to psychological violence and learning of the serious of the consequences for health care workers’ physical and mental health as well as the functioning of the entire health system.

The majority of respondents in our study reported that there were no procedures for reporting violence in their workplace; when there were such procedures, many only reported incidents of physical violence. Although the results were not significant, having procedures for reporting violence is considered a protective factor for WPV by many researchers. However, only having procedures for reporting violence is insufficient; hospital leaders must also encourage employees to report incidents of WPV. The attention of hospital leaders is an important prerequisite of dealing with violence. However, one consequence of a failure to report WPV is an absence of evidence to help health policymakers become aware of WPV.

Only 39.17% of respondents reported having training in the management of aggression and violence in our study. This suggests that training must be sustained at the organizational level in order to prevent and respond to psychological violence. From a management perspective, first, hospital managers should organize medical staff to convene an exchange of medical violence experiences. By pooling experience in this way, hospitals could ensure the early prevention and reduce the harm caused by violence. Second, these managers should assess current riot control measures in their respective health facilities. Finally, they might refer to violence prevention research in other preventive health care institutions [49–52], and to train staff to prevent and respond to hospital violence, such as by teaching emotional conditioning skills to help staff manage patients’ or families’ negative emotions (e.g., medical anger) and or interpersonal communication skills to promote more effective communication between patients and staff.

### Limitations

The present study has several limitations. First, because of time and resource restrictions, our study was limited to 90 purposively selected township hospitals in a single province in China. Therefore, we cannot generalize our findings to all of the township hospitals in Heilongjiang province or all of China. However, our findings might provide a guide for further research on WPV in Chinese township hospitals. Second, this study was retrospective and involved questionnaires that required respondents to recall events occurring in the past 12 months. This makes the data subject to recall bias.

## Conclusions

Township hospitals are important primary health care institutions in China, and it is becoming increasingly important to attend to WPV in these hospitals. The results of this study indicate that there is a high prevalence of psychological violence against health care workers in such hospitals. Considering that more than half of respondents did not report the violence, it is important to establish appropriate reporting systems and provide training programs for health professionals in order to prevent and manage WPV, especially psychological violence. This study found some risk factors of psychological violence among general practitioners and nurses in township hospitals in Heilongjiang Province, which provided a good reference for our policy making and the management of township hospitals to prevent psychological violence in hospitals.However, our extrapolation of our results also requires increasing the sample size or taking into account the specific circumstances of each region. For future research, we would like to assess the effectiveness of current measures to prevent and resolve violence in Chinese township hospitals.

## Abbreviations

GPs: General practitioners
WHO: World Health Organization
WPV: Workplace violence
